# Urate-lowering therapy for CKD patients with asymptomatic hyperuricemia without proteinuria elucidated by attribute-based research in the FEATHER Study

**DOI:** 10.1038/s41598-022-07737-9

**Published:** 2022-03-08

**Authors:** Hiroshi Kataoka, Toshio Mochizuki, Mamiko Ohara, Yuki Tsuruta, Naomi Iwasa, Rie Yoshida, Ken Tsuchiya, Kosaku Nitta, Kenjiro Kimura, Tatsuo Hosoya, the FEATHER Investigators, Kenjiro Kimura, Tatsuo Hosoya, Sadayoshi Ito, Masaaki Inaba, Yasuhiko Tomino, Shunya Uchida, Hirofumi Makino, Seiichi Matsuo, Hisashi Yamanaka, Tetsuya Yamamoto, Iwao Ohno, Yugo Shibagaki, Satoshi Iimuro, Naohiko Imai, Masanari Kuwabara, Hiroshi Hayakawa, Tadao Akizawa, Tamio Teramoto, Hiroshi Kasanuki, Kenichi Yoshimura, Kenjiro Kimura, Tatsuo Hosoya, Yugo Shibagaki, Iwao Ohno, Hiroshi Sato, Shunya Uchida, Satoshi Horikoshi, Syoichi Maruyama, Masahiko Inaba, Yuji Moriwaki, Haruhito Uchida, Nagayuki Kaneshiro, Naohiko Imai, Hidekazu Moriya, Yasuhiro Komatsu, Shinya Kaname, Kazunari Hanaoka, Makoto Ogura, Masato Ikeda, Kenji Kasai, Akira Sugiura, Kazushi Takahashi, Kenichiro Kojima, Kosaku Nitta, Hirofumi Tamai, Hiroshi Nagaya, Senji Okuno, Ryusuke Kakiya, Hiroya Takeoka, Kyouji Hirata, Kenichiro Asano, Yasuo Fukaya, Yasushi Iwaida, Yasuo Tsuneda, Shigeaki Nishimura, Takeyuki Hiramatsu, Yoshitaka Isaka, Takafumi Ito, Yukio Yuzawa, Kunihiro Yamagata, Tadashi Sofue, Yoshimi Jinguji, Keita Hirano, Kazuhiro Matsuyama, Teruhiko Mizumoto, Yuko Shibuya, Masahiro Sugawara, Moritoshi Kadomura, Yasuaki Teshima, Hiroshi Ohtani, Hiroki Kamata, Susumu Okawara, Masaki Fukushima, Katsumi Takemura, Eriko Kinugasa, Masami Kogure, Yoichi Ehara

**Affiliations:** 1 Department of Nephrology, Tokyo Women’s Medical University, Tokyo, 162-8666 Japan; 2 Department of Nephrology, Clinical Research Division for Polycystic Kidney Disease, Tokyo Women’s Medical University, Tokyo, 162-8666 Japan; 3 Department of Nephrology, Kameda Medical Center, Chiba, 296-8602 Japan; 4 Department of Blood Purification, Tokyo Women’s Medical University, Tokyo, 162-8666 Japan; 5 Tokyo Takanawa Hospital, Tokyo, 108-8606 Japan; 6 Division of Chronic Kidney Disease Therapeutics, The Jikei University, Tokyo, Japan; 7 Tohoku University, Sendai, Japan; 8 Osaka City University, Osaka, Japan; 9 Juntendo University, Tokyo, Japan; 10 Teikyo University, Tokyo, Japan; 11 Okayama University, Okayama, Japan; 12 Nagoya University, Nagoya, Japan; 13 Institute of Rheumatology, Tokyo Women’s Medical University, Tokyo, Japan; 14 Hyogo College of Medicine, Nishinomiya, Japan; 15 Jikei University, Tokyo, Japan; 16 St. Marianna University School of Medicine, Kawasaki, Japan; 17 Tokyo Women’s Medical University, Tokyo, Japan; 18 Toranomon Hospital, Tokyo, Japan; 19 Showa University, Tokyo, Japan; 20 Waseda University, Tokyo, Japan; 21 Kanazawa University, Kanazawa, Japan; 22 The Jikei University School of Medicine, Tokyo, Japan; 23 St. Marianna University School of Medicine Hospital, Kawasaki, Japan; 24 Tohoku University Hospital, Sendai, Japan; 25 Teikyo University Hospital, Tokyo, Japan; 26 Juntendo University Hospital, Tokyo, Japan; 27 Nagoya University Hospital, Nagoya, Japan; 28 Osaka City University Hospital, Osaka, Japan; 29 The Hospital of Hyogo College of Medicine, Nishinomiya, Japan; 30 Okayama University Hospital, Okayama, Japan; 31 Kawasaki Municipal Tama Hospital, Kawasaki, Japan; 32 St. Marianna University School of Medicine Yokohama Seibu Hospital, Yokohama, Japan; 33 Shonan Kamakura General Hospital, Kamakura, Japan; 34 St. Luke’s International Hospital, Tokyo, Japan; 35 Kyorin University Hospital, Mitaka, Japan; 36 Jikei University Daisan Hospital, Tokyo, Japan; 37 The Jikei University Kashiwa Hospital, Kashiwa, Japan; 38 The Jikei University Katsushika Medical Center, Tokyo, Japan; 39 Fuji City General Hospital, Fuji, Japan; 40 Osaki Citizen Hospital, Osaki, Japan; 41 Tokyo-Kita Medical Center, Tokyo, Japan; 42 Ageo Central General Hospital, Ageo, Japan; 43 Anjo Kosei Hospital, Anjo, Japan; 44 Yokkaichi Municipal Hospital, Yokkaichi, Japan; 45 Shirasagi Hospital, Osaka, Japan; 46 Meijibashi Hospital, Matsubara, Japan; 47 Hyogo Prefectural Amagasaki General Medical Center, Amagasaki, Japan; 48 Chugoku Central Hospital, Fukuyama, Japan; 49 Kurashiki Central Hospital, Kurashiki, Japan; 50 Southern TOHOKU Research Institute for Neuroscience, Koriyama, Japan; 51 Nippon Kokan Hospital, Kasawaki, Japan; 52 Fujisawa City Hospital, Fujisawa, Japan; 53 Ehime Prefectural Central Hospital, Matsuyama, Japan; 54 Konan Kosei Hospital, Konan, Japan; 55 Osaka University Hospital, Suita, Japan; 56 Shimane University Hospital, Izumo, Japan; 57 Fujita Health University Hospital, Toyoake, Japan; 58 University of Tsukuba Hospital, Tsukuba, Japan; 59 Kagawa University Hospital, Kagawa, Japan; 60 Yamanashi Prefectural Central Hospital, Yamanashi, Japan; 61 Japanese Red Cross Ashikaga Hospital, Tochigi, Japan; 62 Matsuyama Clinic Oita Nephro Medical Clinic, Oita, Japan; 63 Kumamoto General Hospital, Yatsushiro, Japan; 64 NTT Medical Center Tokyo, Tokyo, Japan; 65 Sugawara Clinic, Tokyo, Japan; 66 National Hospital Organization Chiba-East-Hospital, Chiba, Japan; 67 Ikeda Kinen Hospital, Sukagawa, Japan; 68 Iwase General Hospital, Sukagawa, Japan; 69 Medical Corporation Kuon-Kai Kamata Medical Clinic, Tokyo, Japan; 70 Saitama Medical Center Jichi Medical University, Saitama, Japan; 71 Shigei Medical Research Hospital, Okayama, Japan; 72 Takemura Medical Nephro Clinic, Kanuma, Japan; 73 Showa University Northern Yokohama Hospital, Yokohama, Japan; 74 Kogure Clinic, Maebashi, Japan; 75 Yoshii Central Clinic, Takasaki, Japan; 76 Tokyo Women’s Medical University Hospital, Tokyo, Japan

**Keywords:** Diseases, Nephrology

## Abstract

Attribute-based medicine is essential for patient-centered medicine. To date, the groups of patients with chronic kidney disease (CKD) requiring urate-lowering therapy are clinically unknown. Herein, we evaluated the efficacy of febuxostat using a cross-classification, attribute-based research approach. We performed post hoc analysis of multicenter, randomized, double-blind, placebo-controlled trial data for 395 patients with stage 3 CKD and asymptomatic hyperuricemia. Participants were divided into febuxostat or placebo groups and subcohorts stratified and cross-classified by proteinuria and serum creatinine concentrations. In patients stratified based on proteinuria, the mean eGFR slopes were significantly higher in the febuxostat group than in the placebo group (*P* = 0.007) in the subcohort without proteinuria. The interaction between febuxostat treatment and presence of proteinuria in terms of eGFR slope was significant (P for interaction = 0.019). When cross-classified by the presence of proteinuria and serum creatinine level, the mean eGFR slopes significantly differed between the febuxostat and placebo groups (*P* = 0.040) in cross-classified subcohorts without proteinuria and with serum creatinine level ≥ median, but not in the cross-classified subcohorts with proteinuria and serum creatinine level < median. Febuxostat mitigated the decline in kidney function among stage 3 CKD patients with asymptomatic hyperuricemia without proteinuria.

## Introduction

The definition of chronic kidney disease (CKD) has been widely disseminated and generally accepted^1^. However, some controversies remain in CKD research^2,3^, including the effects of nephrosclerosis^4^ and urate-lowering therapy for CKD^5^. Although urate-lowering therapy is not generally approved for patients with asymptomatic hyperuricemia^6^, pharmacological treatment of hyperuricemia in patients with CKD is recommended in Japan^7^.

As risk factors and pathological conditions for CKD progression differ with the etiology and patient attributes, studies are needed to consider these factors. Nephrosclerosis is characterized by the absence of or minimal proteinuria associated with slowly progressing kidney disease^4^, and the number of patients with end-stage kidney disease due to nephrosclerosis is increasing with the aging population^6^. Interestingly, hyperuricemia in patients with CKD has gained attention owing to the global spread of arteriosclerosis^7,8^ and is associated with kidney arteriolopathy^9,10^. Animal studies have reported a direct negative effect of uric acid on endothelial^11^ and smooth muscle cells^12,13^ in kidney arterial/arteriolar vessels. The observation period of several clinical trials was short because of the associated expenses, making it difficult to detect the outcomes of CKD involving slowly progressive nephrosclerotic kidney diseases.

The Febuxostat Versus Placebo Randomized Controlled Trial Regarding Reduced Renal Function in Patients With Hyperuricemia Complicated by Chronic Kidney Disease Stage 3 (FEATHER) study (follow-up, 108 weeks) failed to confirm that febuxostat alleviated the decline in the estimated glomerular filtration rate (eGFR) in stage 3 CKD patients with hyperuricemia without gout in the entire cohort. However, the FEATHER study reported substantial suppression of eGFR decline in patients without proteinuria (*P* = 0.005) or a serum creatinine level lower than the median (*P* = 0.009) after febuxostat treatment^14^.

Patient-centered medicine^15^ and personalized evidence-based medicine (EBM)^16^ have recently garnered attention. Although randomized controlled trials (RCTs) are the cornerstone of EBM with the highest-quality evidence when setting clinical guidelines and policies for patient care^17^, ingenuity in adapting RCT results to individual patients is required^18^. Although subgroup analyses of the heterogeneity of treatment effects will provide valuable information for patient care^15^ and can lead to personalized EBM^16,19^, the characteristics of patients may influence the efficacy of therapy; thus, conventional subgroup analyses are typically not informative^16,20^. This is because RCT results are generally examined through conventional subgroup analyses, contrasting effects in groups of patients, and defining one variable or attribute at a time^18,21^. Such single-attribute comparisons intrinsically result in subcohorts that are more similar to the total cohort because of the averaging of the multiple characteristics of patients in cohorts comprising heterogeneous patients^16,20^. Thus, conventional subgroup (one-attribute-at-a-time) analyses under-represent heterogeneity among patients who differ in several variables^16^.

**Figure 1:**
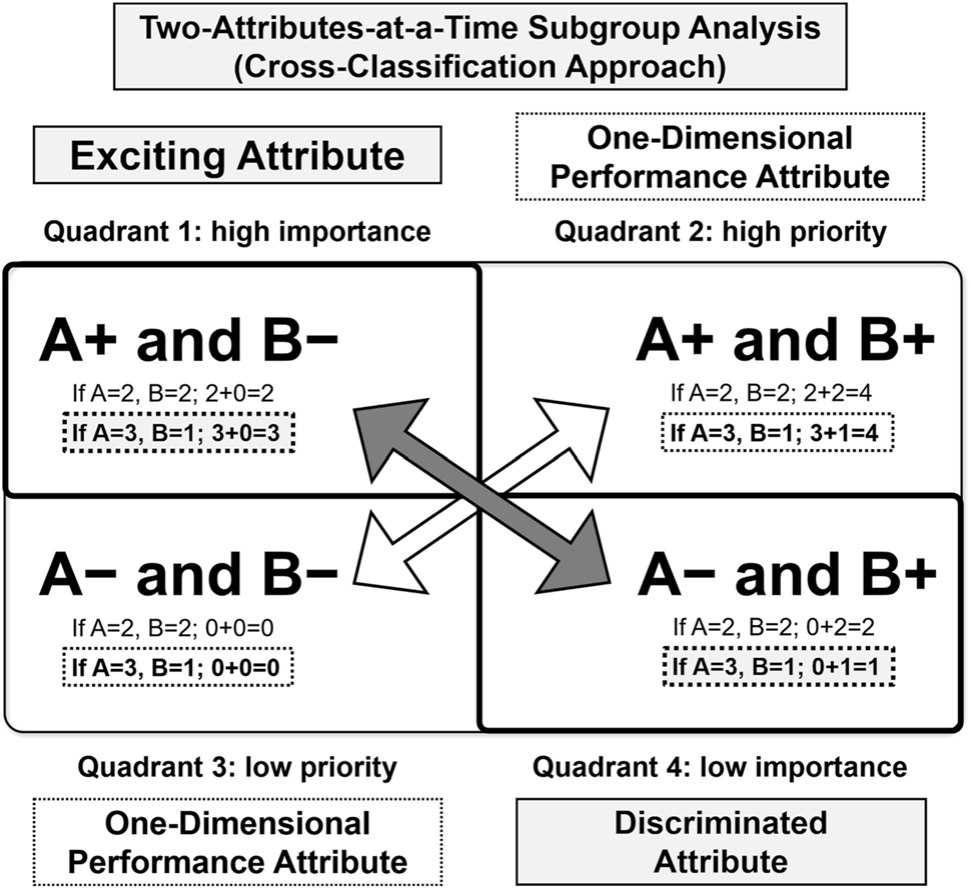
Cross-classification approach for attributes (importance grid analysis). Cross-classification of the attributes showing which attribute, A or B, is important. First, Quadrants 2 and 3, which are diagonal elements, generally become one-dimensional performance attributes with a high value in Quadrant 2 and low value in Quadrant 3. Quadrants 1 and 4, which are off-diagonal elements, can be used to discriminate the importance of A and B. When the attribute value of A is higher than that of B, Quadrant 1 becomes an exciting attribute. For example, if the attribute value of A is 3 and that of B is 1, the attribute value of A positive and B negative is 3, which is higher than Quadrant 4 (A negative and B positive, which has an attribute value of 1). Therefore, by considering differences between Quadrants 1 and 4, the difference in the importance of A and B can be found. A+, A positive; A−, A negative; B+, B positive; B−, B negative.

Two-attributes-at-a-time subgroup analysis (cross-classification approach) is frequently used in research for marketing and has expanded to various fields^22,23^. In cross-classified models, the main diagonal elements are responsible for direct effects and off-diagonal elements are responsible for indirect effects, which are important for discriminating subcohorts^24^. As shown in Fig. 1, a cross-classified structure can help identify interactions between attributes and determine the attribute (A or B) that is important by evaluating the off-diagonal values^24^. The primary outcome in an RCT can be influenced by baseline prognostic factors such as disease severity, referred to as chance bias^25^. Chance bias can be eliminated by stratified randomization or minimization^25^ and by adjusting the imbalance in baseline variables^16,20^. Notably, proteinuria and decreased kidney function are precise indices of the severity of CKD^1^ and major risk factors for kidney disease progression^1,23,26^, which should be adequately adjusted in RCTs in patients with CKD. However, the prognostic factors that affected the efficacy of febuxostat in the FEATHER study are unclear. Furthermore, the need for a plausible hypothesis for treatment heterogeneity between patients with and without proteinuria in urate-lowering therapy for CKD has been increasing^5,27^. Therefore, it is necessary to identify a precise subcohort suitable for febuxostat therapy. We hypothesized that the efficacy of febuxostat is affected by proteinuria, and febuxostat is more useful for populations without proteinuria, comprising patients with atherosclerosis. To overcome the limitations of the conventional one-attribute-at-a-time subgroup analysis and to adjust two major and interactive risk factors in CKD simultaneously, we performed novel cross-classification subgroup analyses of patients in the FEATHER study. We aimed to investigate whether proteinuria or kidney function affects the efficacy of febuxostat.

## Results

### Study patients

The baseline characteristics of the patients are presented in Table 1 and Supplementary Tables S1 and S2. Table 1 presents the overall baseline characteristics of the study participants based on febuxostat use and proteinuria presence. In total, 395 stage 3 CKD patients with hyperuricemia without gout (305 males and 90 females; mean age, 64.7 years; mean eGFR, 45.2 mL/min/1.73 m^2^) were treated with febuxostat (203 patients) or placebo (192 patients). The study groups were well-matched regarding eGFR, sex, age, proteinuria, and diabetes mellitus, with no significant differences in the baseline characteristics from those of the entire cohort. Patients without proteinuria were significantly older (66.4 ± 10.8 vs. 62.6 ± 12.8 years, *P* = 0.002) and had significantly higher eGFR (46.2 ± 9.6 vs. 43.9 ± 9.6 mL/min/1.73 m^2^, *P* = 0.02) than those with proteinuria. Supplementary Table S1 (online) presents the baseline characteristics of study participants for febuxostat treatment stratified by the presence of proteinuria, and Supplementary Table S2 (online) presents the baseline characteristics for febuxostat treatment cross-classified by the presence of proteinuria and the serum creatinine level. The patients’ baseline clinical characteristics and laboratory results were similar among the assigned treatment groups, with no significant differences between both subgroups stratified by the presence of proteinuria and those cross-classified by the presence of proteinuria/serum creatinine level.

**Table 1:** Baseline characteristics of the study participants according to febuxostat treatment and presence or absence of proteinuria.

	Total (n = 395)	Placebo (n = 203)	Febuxostat (n = 192)	*P*-value	Proteinuria-negative (n = 218)	Proteinuria-positive (n = 177)	*P*-value
**Clinical characteristics**							
Male sex	305 (77.2%)	154 (75.9%)	151 (78.6%)	0.510	178 (81.7%)	127 (71.8%)	0.020
Age (years)	64.7 ± 11.9	64.7 ± 12.2	64.7 ± 11.5	0.978	66.4 ± 10.8	62.6 ± 12.8	0.002
Body mass index (kg/m^2^)	24.8 ± 4.1	24.7 ± 3.6	24.9 ± 4.5	0.641	24.8 ± 4.0	24.8 ± 4.2	0.960
Systolic blood pressure (mmHg)	130.6 ± 15.0	129.6 ± 15.0	131.7 ± 15.0	0.170	130.1 ± 15.1	131.1 ± 14.9	0.512
Diastolic blood pressure (mmHg)	77.9 ± 10.8	77.3 ± 11.1	78.4 ± 10.6	0.309	77.2 ± 10.5	78.6 ± 11.2	0.187
Current or former smoker	237 (60.0%)	117 (57.6%)	120 (62.5%)	0.612	127 (58.3%)	110 (62.1%)	0.226
**Laboratory results**							
Estimated GFR (mL/min/1.73 m^2^)	45.2 ± 9.6	45.0 ± 9.8	45.3 ± 9.5	0.775	46.2 ± 9.6	43.9 ± 9.6	0.020
Serum creatinine (mg/dL)	1.26 ± 0.25	1.26 ± 0.25	1.26 ± 0.24	0.880	1.24 ± 0.23	1.28 ± 0.26	0.146
Serum uric acid (mg/dL)	7.96 ± 0.63	7.98 ± 0.64	7.94 ± 0.62	0.539	7.93 ± 0.61	8.00 ± 0.65	0.293
Hemoglobin A1c (%)	6.0 ± 0.6	6.0 ± 0.6	6.0 ± 0.6	0.214	6.0 ± 0.5	6.0 ± 0.6	0.477
UACR (mg/g)	117.0 [17.2–518.0]	120.0 [17.2–517.0]	110.5 [18.1–521.5]	0.958	24.1 [7.1–85.7]	534.0 [224.0–981.0]	< 0.001
Proteinuria	177 (44.8%)	93 (45.8%)	84 (43.8%)	0.680	0 (0.0)	177 (100%)	NA
**Coexisting conditions**							
Diabetes mellitus	114 (28.9%)	59 (29.1%)	55 (28.6%)	0.927	63 (28.9%)	51 (28.8%)	0.985
Ischemic heart disease	24 (6.1%)	11 (5.4%)	13 (6.8%)	0.574	14 (6.4%)	10 (5.6%)	0.749
Cerebrovascular disease	43 (10.9%)	17 (8.4%)	26 (13.5%)	0.099	25 (11.5%)	18 (10.2%)	0.680
**Medications**							
ACE inhibitor and/or ARB	310 (78.5%)	152 (74.9%)	158 (82.3%)	0.073	150 (68.8%)	160 (90.4%)	< 0.001
Statins	150 (38.0%)	68 (33.5%)	82 (42.7%)	0.059	70 (32.1%)	80 (45.2%)	0.008
Antidiabetic drugs	81 (20.5%)	45 (22.2%)	36 (18.8%)	0.400	44 (20.2%)	37 (20.9%)	0.860
Diuretics	71 (18.0%)	32 (15.8%)	39 (20.3%)	0.239	40 (18.3%)	31 (17.5%)	0.830

Values for categorical variables are given as count (percentage); values for continuous variables as mean ± standard deviation; non-normally distributed data as median [quartile 1–quartile 3].

*ACE* angiotensin-converting enzyme, *ARB* angiotensin receptor blocker, *CKD* chronic kidney disease, *GFR* glomerular filtration rate, *UACR* urinary albumin-creatinine ratio.

### Primary endpoints

The eGFR slope analysis results are presented in Table 2. During observation for 108 weeks, statistical analysis of the population mean eGFR slope values of individual patients revealed no significant differences between the febuxostat and placebo groups (between-group difference, 0.31 mL/min/1.73 m^2^/year; *P* = 0.76). However, when stratified by proteinuria, the mean eGFR slope values significantly differed between the febuxostat and placebo groups (between-group difference, 0.72 mL/min/1.73 m^2^/year; *P* = 0.007) in the subcohort without proteinuria. The interaction between febuxostat treatment and the presence of proteinuria in terms of eGFR slope was significant (*P* for interaction = 0.02). When stratified by serum creatinine level, a similar tendency was observed: the mean eGFR slopes differed between the febuxostat and placebo groups (between-group difference, 0.53 mL/min/1.73 m^2^/year; *P* = 0.06) in the subcohort with serum creatinine level < median.

**Table 2:** Analysis of the eGFR slopes.

Cohort	Group	N	Mean	Between-group difference, mL/min/1.73 m^2^/year (95% CI)	*P*	*P* for interaction
Entire cohort	Placebo	203	− 0.46	0.31 (− 0.11 to 0.73)	0.762	NA
	Febuxostat	192	− 0.15			
Entire cohort	Proteinuria-negative	218	0.20	− 1.15 (− 1.56 to − 0.74)	< 0.001	NA
	Proteinuria-positive	177	− 0.94			
Entire cohort	s-Cre < median	192	0.19	− 0.98 (− 1.39 to − 0.57)	< 0.001	NA
	s-Cre ≥ median	203	− 0.79			
**Proteinuria**						0.019
Negative	Placebo	110	− 0.15	0.72 (− 0.20 to 1.24)	0.007	
	Febuxostat	108	0.60			
Positive	Placebo	93	− 0.82	− 0.25 (− 0.89 to 0.39)	0.439	
	Febuxostat	84	− 1.08			
**s-Cre**						0.283
< Median	Placebo	98	− 0.07	0.53 (− 0.02 to 1.09)	0.061	
	Febuxostat	94	0.47			
≥ Median	Placebo	105	− 0.83	0.09 (− 0.52 to 0.69)	0.780	
	Febuxostat	98	− 0.74			
**Cross-classified cohort**						0.064
Proteinuria-negative and s-Cre < median	Placebo	52	0.45	0.64 (− 0.09 to 1.38)	0.086	
	Febuxostat	55	1.09			
Proteinuria-negative and s-Cre ≥ median	Placebo	58	− 0.69	0.72 (0.03 to 1.41)	0.040	
	Febuxostat	53	0.03			
Proteinuria-positive and s-Cre < median	Placebo	46	− 0.65	− 0.24 (− 0.54 to 1.01)	0.543	
	Febuxostat	39	− 0.41			
Proteinuria-positive and s-Cre ≥ median	Placebo	47	− 0.99	− 0.65 (− 0.33 to 1.64)	0.191	
	Febuxostat	45	− 1.65			

*CI* confidence interval, *eGFR* estimated glomerular filtration rate, *s-Cre* serum creatinine.

**Figure 2:**
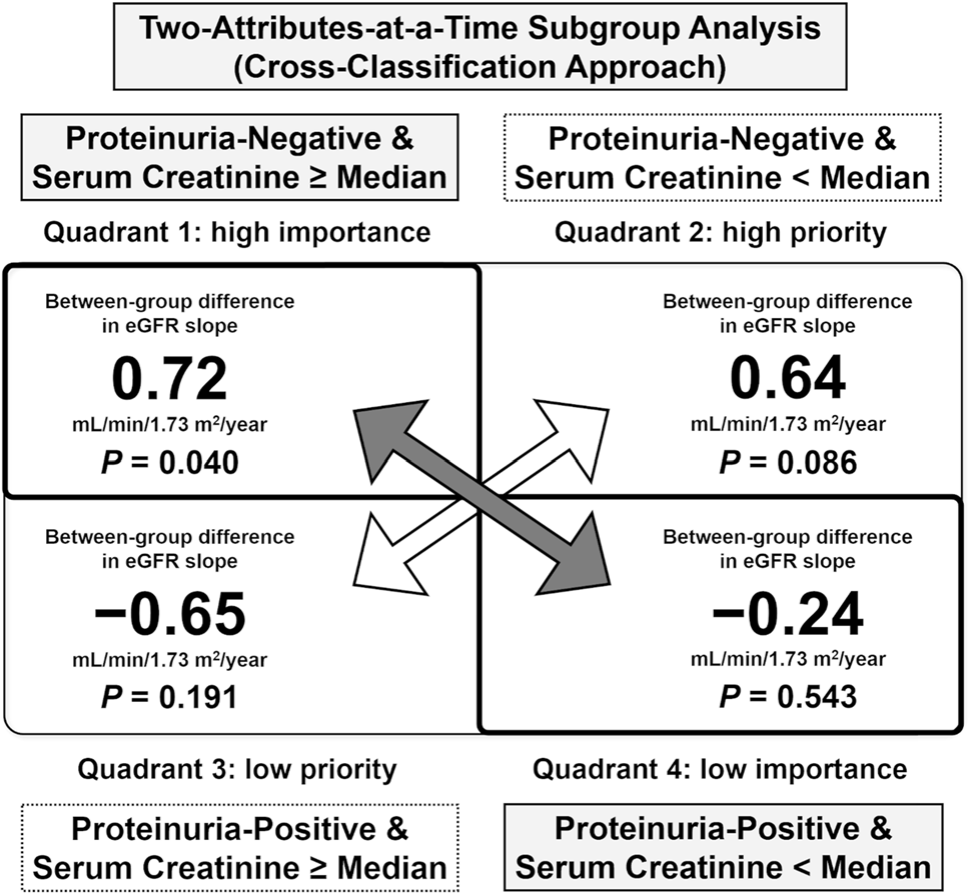
Cross-classification approach (importance grid analysis) for attributes regarding between-group differences in the estimated glomerular filtration rate (eGFR) slope. Quadrant 1: without proteinuria and serum creatinine level ≥ median; Quadrant 2: without proteinuria and serum creatinine level < median; Quadrant 3: with proteinuria and serum creatinine level ≥ median; Quadrant 4: with proteinuria and serum creatinine level < median. Quadrants 1 and 4 can be used to discriminate the importance of the absence of proteinuria and a serum creatinine level < median for the efficacy of febuxostat in patients with chronic kidney disease. As the between-group difference in the eGFR slope in quadrant 1 was significant for the efficacy of febuxostat, despite the fact that the serum creatinine level was ≥ median, the absence of proteinuria is a more important attribute compared to the serum creatinine level being < median. Accordingly, the absence of proteinuria is an attribute affecting the efficacy of febuxostat in patients with chronic kidney disease (all values were derived from Table 1).

When cross-classified by proteinuria and serum creatinine level, the mean eGFR slopes significantly differed between the febuxostat and placebo groups (between-group difference, 0.72 mL/min/1.73 m^2^/year; *P* = 0.04) in the cross-classified subcohorts without proteinuria and with the serum creatinine level ≥ median, but not in the cross-classified subcohorts with proteinuria and serum creatinine level < median (between-group difference, − 0.24 mL/min/1.73 m^2^/year; *P* = 0.5), indicating that the presence of proteinuria exhibited an association stronger than a high serum creatinine level with febuxostat efficacy (Fig. 2). Interactions between febuxostat treatment and a proteinuria/serum creatinine level ≥ median in terms of the eGFR slope were significant (*P* for interaction = 0.06).

### Secondary endpoints

**Figure 3:**
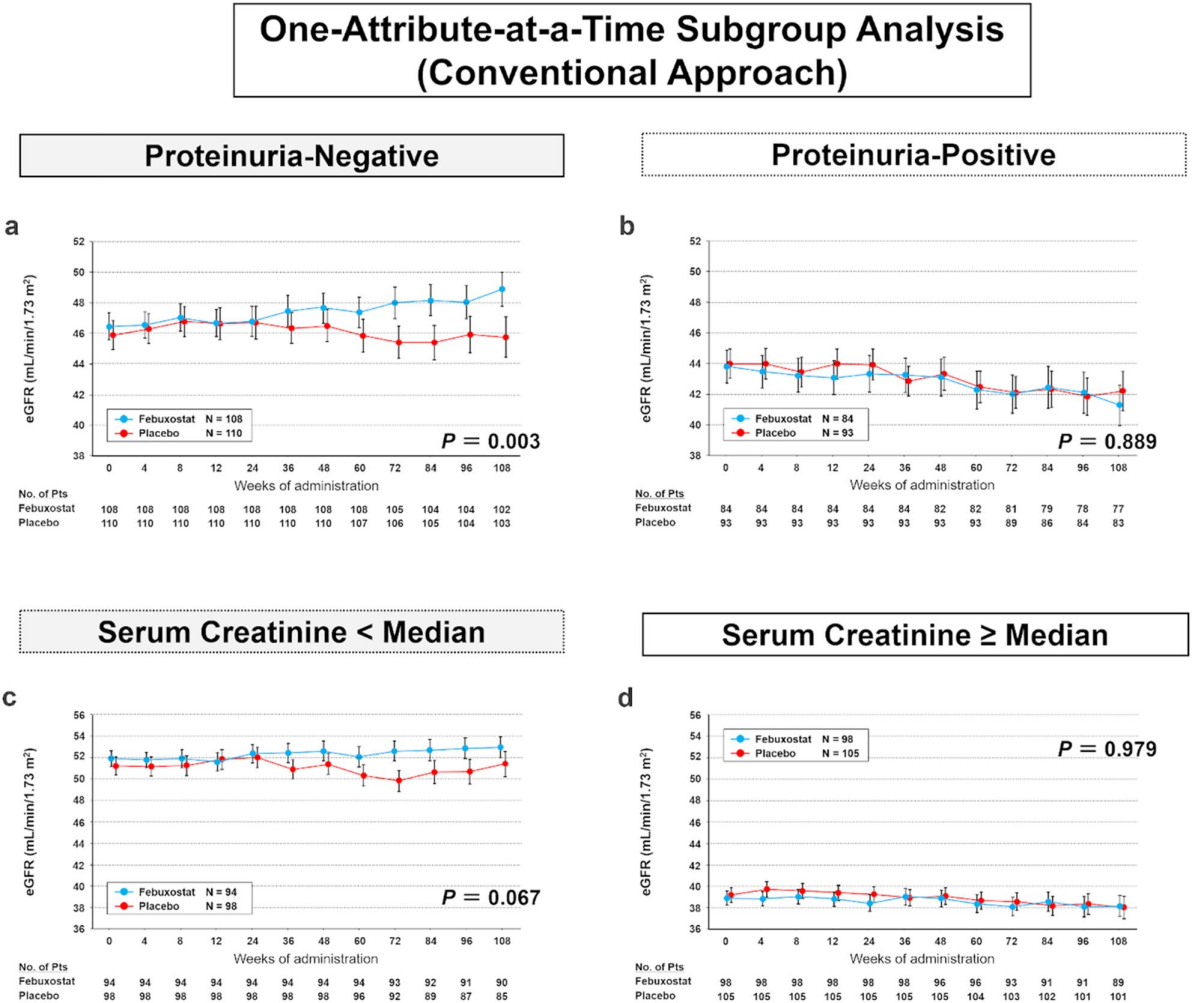
Time-course changes in the estimated glomerular filtration rates (eGFRs) from week 0 through week 108 of treatment. (**a**) Subcohort of patients without proteinuria. (**b**) Subcohort of patients with proteinuria. (**c**) Subcohort of patients with serum creatinine level < median. (**d**) Subcohort of patients with serum creatinine level ≥ median. The mean eGFR in the two groups is shown at different timepoints during the trial. Error bars indicate the standard error. The mean eGFR is shown for participants with available levels at each timepoint. *P*-values were obtained by a test of the trend profile using a mixed model.

Supplementary Figs. S2 and S3 show time-course changes in the eGFRs from weeks 0 to 108 of febuxostat treatment. Between-group differences in the mean eGFR increased at week 24 or later in the entire cohort (Supplementary Fig. S2) and significantly increased in the subcohort without proteinuria (Fig. 3a, P = 0.003); however, this increase was not observed in the subgroup with proteinuria (Fig. 3b). When stratified by the serum creatinine level, between-group differences in the mean eGFR increased in the subcohort with a serum creatinine level < median (Fig. 3c), which was not observed in the subgroup with a serum creatinine level ≥ median (Fig. 3d).

**Figure 4:**
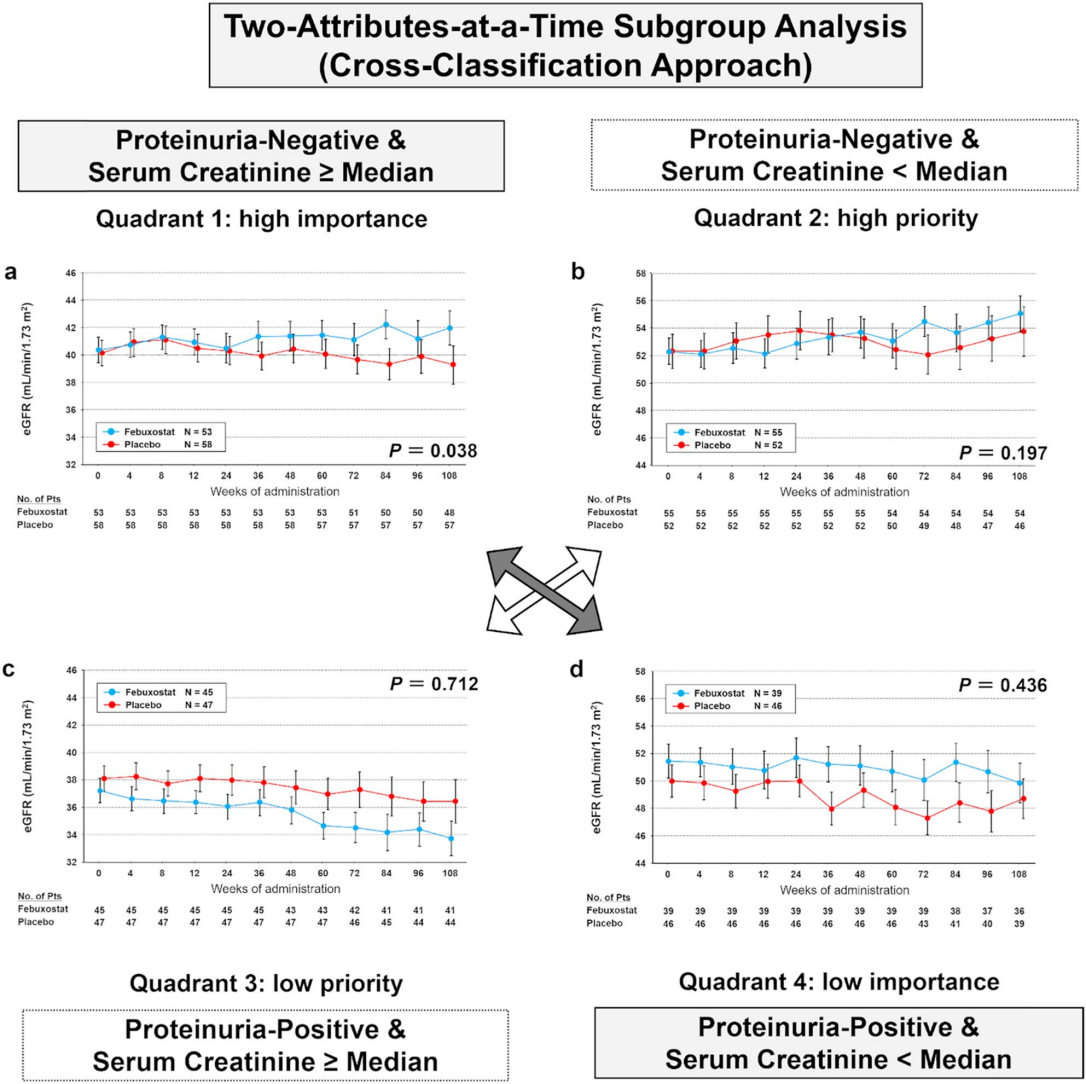
Time-course changes in estimated glomerular filtration rates (eGFRs) from week 0 through week 108 of treatment in subcohorts according to cross-classification. (**a**) Quadrant 1: without proteinuria and a serum creatinine level ≥ median. (**b**) Quadrant 2: without proteinuria and a serum creatinine level < median. (**c**) Quadrant 3: with proteinuria and a serum creatinine level ≥ median. (**d**) Quadrant 4: with proteinuria and a serum creatinine level < median. The mean eGFR in the two groups is shown at different timepoints during the trial. Error bars indicate the standard error. The mean eGFR is shown for participants with available levels at each timepoint. *P*-values were obtained by a test of the trend profile using a mixed model. Similar to Fig. 2, quadrants 1 and 4 can be used to discriminate the importance of the absence of proteinuria and a serum creatinine level < median for the efficacy of febuxostat in patients with chronic kidney disease. As the trend profile in the time-course changes in the eGFRs in quadrant 1 was significant for the efficacy of febuxostat, despite the fact that the serum creatinine level was ≥ median, the absence of proteinuria is a more important attribute compared to the serum creatinine level being < median. Accordingly, the absence of proteinuria is an attribute affecting the efficacy of febuxostat in patients with chronic kidney disease.

When cross-classified by the presence of proteinuria and serum creatinine level (Fig. 4), between-group differences in the mean eGFR significantly increased in the cross-classified subcohorts without proteinuria and serum creatinine level ≥ median (Fig. 4a, P = 0.04). In contrast, an increase was not observed in subcohorts with proteinuria and serum creatinine level < median (Fig. 4d), which was similar to the primary endpoint. These results indicate that the presence of proteinuria is more crucial than the serum creatinine level for febuxostat efficacy.

## Discussion

The pathophysiology and progression of CKD vary substantially across certain subcohorts based on the patients’ proteinuria status, diabetes status, CKD stage, and ethnicities^1,23,28^. Thus, the prognosis for slowly progressive kidney disease caused by hyperuricemia with febuxostat treatment should be assessed in cohorts presenting similar conditions, etiology, and pathophysiology. In the present post hoc analysis of the FEATHER study results, we clarified the differences between patients with and without proteinuria in terms of the efficacy of febuxostat in patients with stage 3 CKD using a cross-classification approach. To the best of our knowledge, this is the first study to elucidate that the presence of proteinuria rather than the serum creatinine level affects the efficacy of febuxostat. Febuxostat was useful for patients with stage 3 CKD without proteinuria, and its efficacy was confirmed even in the subcohort with a serum creatinine level ≥ median.

Recently, an RCT in patients with stage 3–4 CKD (Controlled Trial of Slowing of Kidney Disease Progression from the Inhibition of Xanthine Oxidase trial: CKD-FIX; follow-up, 104 weeks) failed to confirm the benefits of allopurinol on kidney outcomes^29^. However, an RCT does not provide a basis for extrapolation beyond the involved etiology, period, or drugs. The major differences between the present post hoc FEATHER study and the CKD-FIX study were the CKD stage (stage 3 vs. stages 3–4), eGFR (45.2 vs. 31.7 mL/min/1.73 m^2^), eGFR slope in the placebo group (− 0.31 vs. − 3.33 mL/min/1.73 m^2^/year), urinary albumin-to-creatinine ratio (117.0 vs. 716.9 mg/g), proportion of patients with diabetes (29% vs. 45%), ethnicity of the participants (100% Asian vs. 5% Asian), and the agent used (febuxostat vs. allopurinol). The major reason for the difference in efficacy of urate-lowering agents for patients with CKD is unknown, but may involve the presence or absence of proteinuria.

Proteinuria is an important risk factor for kidney diseases, and several patients with glomerulonephritis and diabetic nephropathy present with proteinuria^26^. However, in clinical settings, patients without proteinuria show progressive kidney diseases^30^ and develop end-stage renal disease. Most of these patients are clinically considered to have nephrosclerosis, which shows a slow progression of kidney dysfunction and is referred to as benign nephrosclerosis^31^. However, it has been recently shown that patients with nephrosclerosis have a poor long-term prognosis^32^. Nephrosclerosis is pathologically characterized as arterionephrosclerosis and subsequent tubulointerstitial inflammation/fibrosis. Arterionephrosclerosis is considered associated with systemic atherosclerosis. It arises because of various risk factors, including obesity, oxidative stress, and chronic inflammation, and has two pathological populations^4,31^. One is interstitial inflammatory fibrosis caused by ischemia/hypoxia/oxidative stress^33^ and the other is glomerular hypertrophy caused by losing autoregulation of renal blood flow resulting from an impairment of the afferent arteriole myogenic response^31,34^.

Notably, hyperuricemia has been associated with arteriosclerosis and arterionephrosclerosis/arteriolopathy in human^9,10^ and animal^13,35,36^ studies. Experimental studies of serum and intracellular hyperuricemic models have found that hyperuricemia causes renal arterionephrosclerosis/arteriolopathy owing to the activation of the renin–angiotensin–aldosterone system^35,37^ and decreased nitric oxide bioavailability^35,37^. This results in ischemic injury with tubulointerstitial inflammation/fibrosis^12,35,37^ caused by impaired renal ischemia/hypoxia/intrarenal oxidative stress, leading to glomerular hypertrophy^12,38^. Thus, hyperuricemia is considered to directly and indirectly cause nephrosclerosis.

Febuxostat, a non-purine selective inhibitor of xanthine oxidoreductase (XOR), inhibits both the reduced and oxidized forms of XOR and has been reported to attenuate experimental atherosclerosis in mice^39^. Xanthine oxide (XO) and XOR, both sources of reactive oxygen species, are associated with arteriolopathy and tubulointerstitial inflammation/fibrosis^40^. In animal models, febuxostat has been found to attenuate kidney injuries caused by impaired renal ischemia/hypoxia/intrarenal oxidative stress^12,41^, such as renal arterionephrosclerosis/arteriolopathy^12,41,42^, tubulointerstitial inflammation/fibrosis^12,41,42^, and glomerular hypertrophy^12^. Thus, hyperuricemia and increased XO and XOR activities in animal models result in arteriolopathy and tubulointerstitial inflammation/fibrosis, which is sometimes associated with arteriosclerosis-related diseases without excessive proteinuria; these data are consistent with the findings of human observational studies^10,43^. Therefore, febuxostat is expected to be effective for such conditions in humans^44^, and to the best of our knowledge, the present study is the first to show the effects of febuxostat in patients without proteinuria after six months. The reason for the lack of a febuxostat effect in less than six months is unclear, but may be related to variable glomerular size^31,45,46^. There are two types of nephrosclerosis, glomerular collapse and glomerular hypertrophy^4,31^, and the response to febuxostat may differ at individual nephron levels. Some glomeruli may increase the single-nephron GFR by improving ischemia and some glomeruli may decrease it by improving glomerular hyperfiltration/hypertrophy. Because of this difference, it may take six months for the average renoprotective effect of febuxostat to be detected. Furthermore, improvements in chronic tubular interstitial injury due to renal ischemia/hypoxia/intrarenal oxidative stress may require a long recovery time to restore the tissue damage^47^.

In RCTs for CKD, substantial and identifiable heterogeneity of risk in the trial population is pathophysiologically anticipated owing to the nature of the kidney disease, which can cause a chance bias^16,25^; therefore, we consider that the analysis should be adjusted for baseline attributes^25^. Patient-centered medicine and research have recently attracted attention^15^. In patient-centered research, it is important to evaluate a homogeneous patient group to overcome individual heterogeneity in human studies. Attribute-based studies of homogeneous patient groups are essential for patient-centered medicine, and the two-attributes-at-a-time subgroup analysis (cross-classification approach) can harmonize with RCTs in patients with CKD. Thus, the cross-classification approach will expand and contribute to subgroup analyses in future RCTs.

This study had limitations. As described in a previous study^14^, patients with stage 4 or 5 CKD were excluded. The number of patients was relatively low, and there were more male participants than female participants (males accounted for nearly four-fifths of the entire cohort). Since we adjusted the baseline characteristics using proteinuria and the serum creatinine level (which were confirmed using their interactions with kidney outcome in the original FEATHER study [RCT for CKD]), the attributes for which interaction had not been confirmed, including diuretics, were not completely adjusted. The proportion of patients using diuretics in this study was 18%, which is considered to have little effect on the results. However, the effect of diuretic-related hyperuricemia on kidney outcome remains to be studied. Patients without proteinuria were likely to have clinical nephrosclerosis; however, those who participated in this study were not diagnosed by kidney biopsy. Finally, the association between hyperuricemia and kidney outcomes may not be generalizable to other populations, as all participants were Japanese.

In conclusion, the cross-classification approach revealed that the presence or absence of proteinuria rather than the level of serum creatinine affects febuxostat efficacy. Compared with the placebo, febuxostat mitigated the decline in kidney function among patients with stage 3 CKD asymptomatic hyperuricemia without proteinuria.

## Methods

### Study design

We conducted a post hoc analysis of the results of the FEATHER study, which was a multicenter, randomized, double-blind, parallel-group, placebo-controlled, prospective cohort study in Japan. Patients were enrolled between November 2012 and January 2014, and their last visits were between March 2013 and February 2016. The FEATHER study was registered at the University Hospital Medical Information Network Clinical Trials Registry under UMIN000008343. The design and methods of the FEATHER study have been published previously^14,48^. The study protocol was approved by the ethics committee of each participating medical institution (Tokyo Women’s Medical University, No. 130902), and the study was conducted in accordance with the tenets of the Declaration of Helsinki. The pre-specified subgroups were based on the level of kidney function (median serum creatinine level determined in the primary FEATHER study) and presence or absence of proteinuria.

### Participants

The screening criteria in the FEATHER study have been described previously^48^. In brief, the major inclusion criteria were age > 20 years, hyperuricemia (uric acid concentration > 7.0–10.0 mg/dL), stage 3 CKD, no history of gout, and patients agreed to provide written informed consent. The major exclusion criteria were uncontrolled diabetes mellitus or blood pressure and ≥ 50% variation in serum creatinine concentration within 12 weeks before eligibility confirmation. After screening in the primary FEATHER study, 443 patients from 55 nephrology centers were randomly assigned to the febuxostat or placebo group, and the data of 441 eligible patients were analyzed^14^. In this study, to adjust for an imbalance in the follow-up, 46 individuals who were observed for < 1 year and < 4 timepoints were excluded from the study cohort by the enrollment center (the Japan Clinical Research Support Unit, a not-for-profit organization in Tokyo, Japan). The data of the remaining 395 patients were distributed to an independent statistical expert for analyses (Supplementary Fig. S1).

### Study endpoint

The primary endpoint was the eGFR slope (mL/min/1.73 m^2^/year) or annual change in the eGFR calculated using a linear mixed-effects model with repeated GFR estimates over time for each patient. Mean values and their standard errors for eGFR were also examined from baseline to week 108 to test the trend using a mixed model.

### Statistical analysis

Data are presented as the mean ± standard deviation, median with interquartile range, or proportion. Group differences were evaluated using unpaired Student’s *t*-test, Wilcoxon rank-sum test, chi-square test, or Fisher’s exact test. Time-course changes in the eGFR from baseline through week 108 in the study population and subcohorts were analyzed by testing the trend profile using a mixed model. Analysis of the inconsistency in febuxostat treatment effects in pre-specified subgroups was explored regarding the primary endpoints by adding interaction terms to the generalized linear model. Statistical significance was set at *P* < 0.05. An independent statistical data center (STATZ Institute, Tokyo, Japan) performed statistical analyses using SAS ver. 9.4 software (SAS Institute, Cary, NC, USA).

## Supplementary Information

Supplementary Information.

## Data Availability

The data underlying this article will be shared on reasonable request to the corresponding author.
