# Supplementary material for: Urate-lowering therapy for CKD patients with asymptomatic hyperuricemia without proteinuria elucidated by attribute-based research in the FEATHER Study
Source: Sci Rep. 2022 Mar 8;12:3784. doi: 10.1038/s41598-022-07737-9 (PMC8904814; doi:10.1038/s41598-022-07737-9)
Supplement: Supplementary file 1 — Supplementary Information. [file 41598_2022_7737_MOESM1_ESM.pdf]

Supporting information to "Urate-lowering Therapy for Patients with Asymptomatic Hyperuricemia without Proteinuria Elucidated by Attribute-Based Research in the FEATHER Study"

This supplementary information provides further methodological detail and results for the main paper.

Supporting information

S1 Figure. Flow chart of patient selection.

S2 Figure. Time-course changes in the estimated glomerular filtration rates (eGFRs) from week 0 through week 108 of treatment in the study population.

S1 Table. Baseline characteristics of the study participants receiving febuxostat stratified by the presence or absence of proteinuria.

S2 Table. Baseline characteristics of the study participants receiving febuxostat cross-classified by the presence or absence of proteinuria and serum creatinine level.

S1 Figure. Flow chart of patient selection.

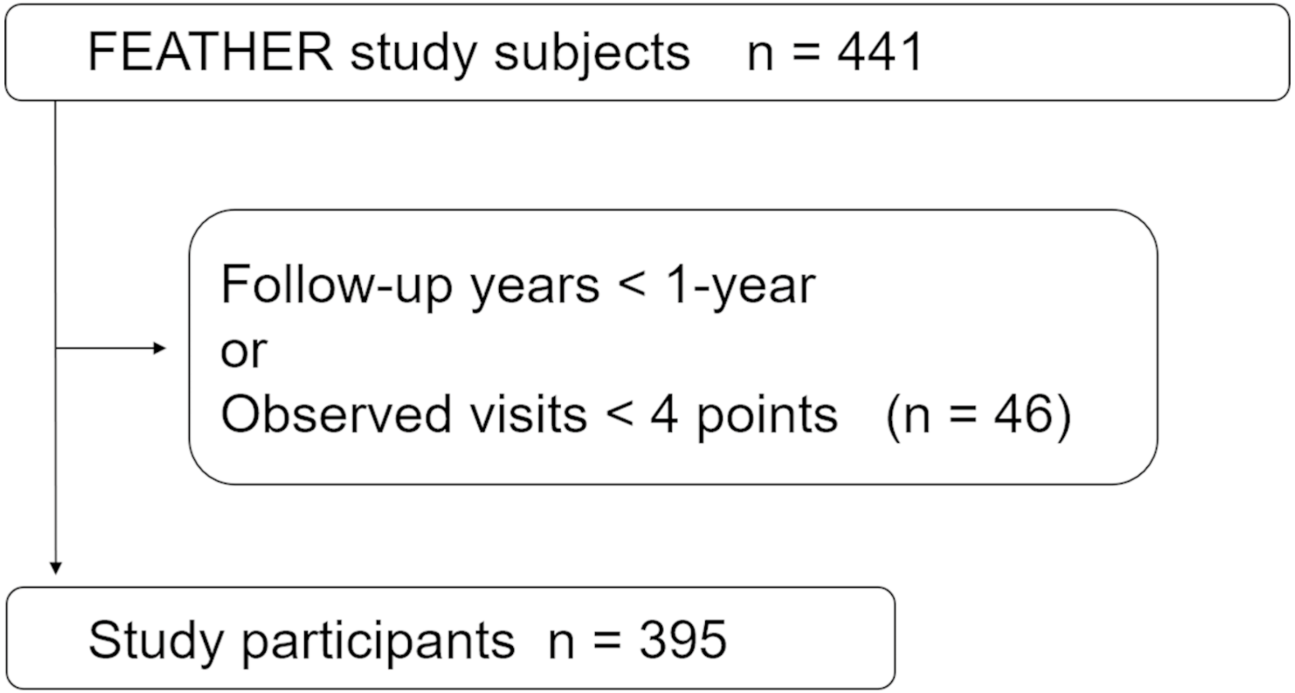

S2 Figure. Time-course changes in the estimated glomerular filtration rates (eGFRs) from weeks 0 through 108 of treatment in the study population.

The mean eGFR level in the two groups are shown at different timepoints during the trial. Error bars indicate standard error. The mean eGFR values are shown for participants with available levels at each timepoint. *P*-values were obtained by a test of trend profile using a mixed model.

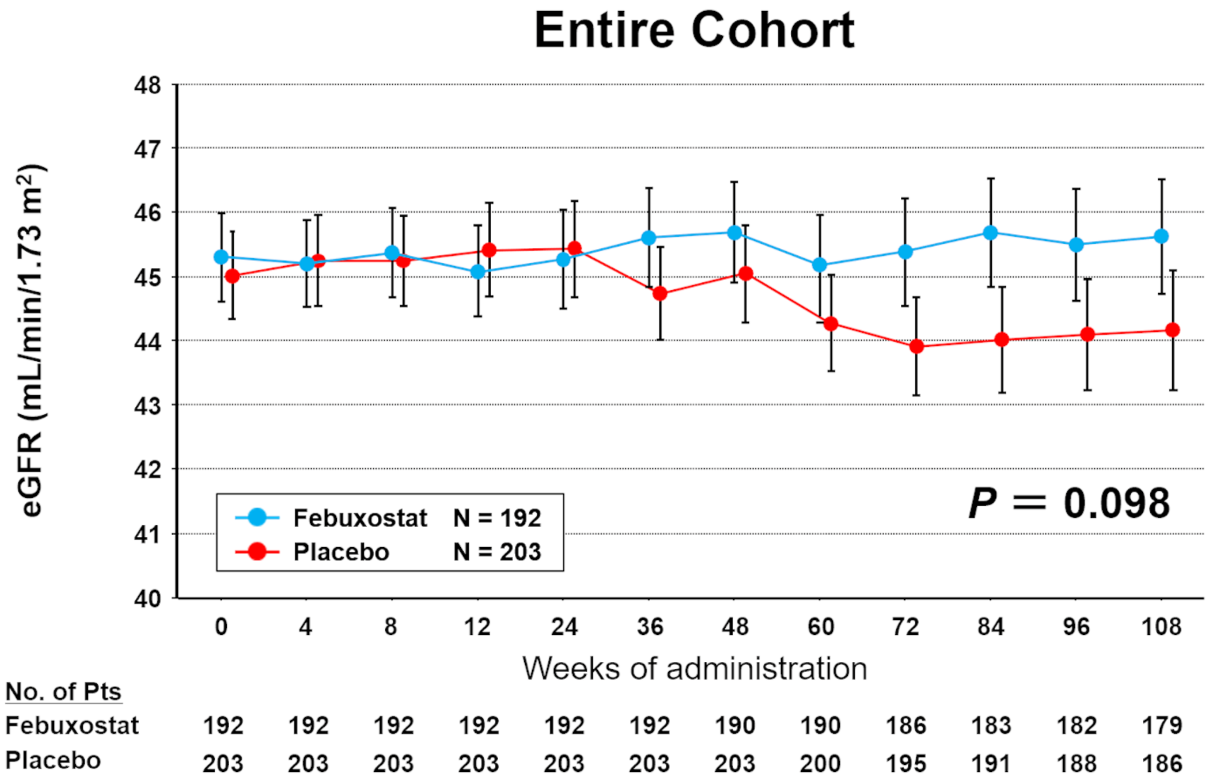

S1 Table. Baseline characteristics of the study participants receiving febuxostat stratified by the presence or absence of proteinuria.

	Proteinuria-Negative (n = 218)			Proteinuria-Positive (n = 177)		
	**Placebo (n = 110)**	**Febuxostat (n = 108)**	***P*–value**	**Placebo (n = 93)**	**Febuxostat (n = 84)**	***P*–value**
**Clinical Characteristics**						
Male sex	89 (80.9%)	89 (82.4%)	0.775	65 (69.9%)	62 (73.8%)	0.563
Age, years	66.6 ± 11.3	66.1 ± 10.2	0.739	62.4 ± 12.9	62.8 ± 12.8	0.854
Body mass index, kg/m^2	24.4 ± 3.6	25.1 ± 4.4	0.196	25.0 ± 3.7	24.5 ± 4.7	0.490
Systolic blood pressure, mmHg	129.5 ± 15.5	130.8 ± 14.8	0.540	129.7 ± 14.5	132.8 ± 15.3	0.164
Diastolic blood pressure, mmHg	77.0 ± 11.4	77.4 ± 9.6	0.795	77.7 ± 10.8	79.8 ± 11.7	0.217
Current or former smoker	60 (54.5%)	67 (62.0%)	0.524	57 (61.3%)	53 (63.1%)	0.948
**Laboratory Results**						
Estimated GFR, mL/min/1.73 m^2	45.9 ± 10.1	46.5 ± 9.1	0.670	44.0 ± 9.3	43.8 ± 9.8	0.907
Serum creatinine, mg/dL	1.25 ± 0.23	1.24 ± 0.23	0.604	1.27 ± 0.27	1.30 ± 0.26	0.448
Serum uric acid, mg/dL	7.95 ± 0.63	7.92 ± 0.60	0.730	8.02 ± 0.66	7.97 ± 0.65	0.621
Hemoglobin A1c, %	6.0 ± 0.5	6.0 ± 0.6	0.820	6.1 ± 0.7	5.9 ± 0.5	0.139
UACR, mg/g	21.2 [7.1-76.7]	24.7 [7.2-87.6]	0.889	508.0 [210.0-973.0]	589.0 [253.0-1060.0]	0.504
**Coexisting conditions**						
Diabetes mellitus	32 (29.1%)	31 (28.7%)	0.950	27 (29.0%)	24 (28.6%)	0.946
Ischemic heart disease	4 (3.6%)	10 (9.3%)	0.090	7 (7.5%)	3 (3.6%)	0.255
Cerebrovascular disease	11 (10.0%)	14 (13.0%)	0.492	6 (6.5%)	12 (14.3%)	0.085
**Medications**						
ACE inhibitor and/or ARB	68 (61.8%)	82 (75.9%)	0.025	84 (90.3%)	76 (90.5%)	0.972
Statins	30 (27.3%)	40 (37.0%)	0.123	38 (40.9%)	42 (50.0%)	0.222
Antidiabetic drugs	22 (20.0%)	22 (20.4%)	0.946	23 (24.7%)	14 (16.7%)	0.188
Diuretics	13 (11.8%)	27 (25.0%)	0.012	19 (20.4%)	12 (14.3%)	0.283

Note: Values for categorical variables are given as count (percentage); values for continuous variables, as mean ± standard deviation; non-normally distributed data, as median [quartile 1-quartile 3].

Abbreviations: ACE, angiotensin-converting enzyme; ARB, angiotensin receptor blocker; CKD, chronic kidney disease; GFR, glomerular filtration rate; UACR, urinary albumin-creatinine ratio.

S2 Table. Baseline characteristics of the study participants receiving febuxostat cross-classified by the presence or absence of proteinuria and serum creatinine level.

	Proteinuria-Negative and s-Cre < Median (n = 107)			Proteinuria-Positive and s-Cre < Median (n = 85)		
	**Placebo (n = 52)**	**Febuxostat (n = 55)**	***P*–value**	**Placebo (n = 46)**	**Febuxostat (n = 39)**	***P*–value**
**Clinical Characteristics**						
Male sex	35 (67.3%)	39 (70.9%)	0.687	25 (54.3%)	27 (69.2%)	0.161
Age, years	66.8 ± 11.4	64.4 ± 10.5	0.254	63.7 ± 12.5	63.5 ± 11.1	0.949
Body mass index, kg/m^2	24.5 ± 4.3	25.4 ± 4.9	0.296	25.2 ± 3.5	25.1 ± 4.7	0.940
Systolic blood pressure, mmHg	131.2 ± 16.9	130.7 ± 14.6	0.874	126.7 ± 14.5	132.9 ± 14.6	0.052
Diastolic blood pressure, mmHg	76.2 ± 11.7	77.3 ± 9.2	0.577	78.0 ± 10.9	79.1 ± 11.5	0.653
Current or former smoker	26 (50.0%)	33 (60.0%)	0.412	28 (60.9%)	22 (56.4%)	0.881
**Laboratory Results**						
Estimated GFR, mL/min/1.73 m^2	52.3 ± 8.9	52.3 ± 7.0	0.991	50.0 ± 8.0	51.4 ± 7.6	0.403
Serum creatinine, mg/dL	1.06 ± 0.10	1.06 ± 0.11	0.833	1.05 ± 0.11	1.07 ± 0.12	0.408
Serum uric acid, mg/dL	7.89 ± 0.61	7.88 ± 0.61	0.892	7.87 ± 0.65	7.94 ± 0.66	0.621
Hemoglobin A1c, %	6.0 ± 0.5	6.0 ± 0.6	0.791	6.0 ± 0.7	5.9 ± 0.4	0.645
UACR, mg/g	16.0 [6.8-68.3]	26.6 [8.7-89.4]	0.503	507.0 [229.0-973.0]	479.0 [212.0-976.0]	0.926
**Coexisting conditions**						
Diabetes mellitus	17 (32.7%)	18 (32.7%)	0.997	10 (21.7%)	10 (25.6%)	0.673
Ischemic heart disease	1 (1.9%)	6 (10.9%)	0.060	5 (10.9%)	0 (0.0)	0.034
Cerebrovascular disease	2 (3.8%)	2 (3.6%)	0.954	3 (6.5%)	7 (17.9%)	0.103
**Medications**						
ACE inhibitor and/or ARB	25 (48.1%)	43 (78.2%)	0.001	43 (93.5%)	35 (89.7%)	0.533
Statins	17 (32.7%)	19 (34.5%)	0.839	17 (37.0%)	19 (48.7%)	0.274
Antidiabetic drugs	12 (23.1%)	13 (23.6%)	0.946	7 (15.2%)	6 (15.4%)	0.983
Diuretics	7 (13.5%)	32 (27.3%)	0.077	9 (19.6%)	5 (12.8%)	0.403

	Proteinuria-Negative and s-Cre ≥ Median (n = 111)			Proteinuria-Positive and s-Cre ≥ Median (n = 92)		
	Placebo (n = 58)	Febuxostat (n = 53)	*P*–value	Placebo (n = 47)	Febuxostat (n = 45)	*P*–value
**Clinical Characteristics**						
Male sex	54 (93.1%)	50 (94.3%)	0.789	40 (85.1%)	35 (77.8%)	0.365
Age, years	66.4 ± 11.3	67.9 ± 9.7	0.460	61.2 ± 13.2	62.2 ± 14.3	0.743
Body mass index, kg/m^2	24.4 ± 2.8	24.8 ± 3.8	0.482	24.7 ± 3.8	24.0 ± 4.6	0.412
Systolic blood pressure, mmHg	128.0 ± 14.2	130.8 ± 15.1	0.311	132.6 ± 14.0	132.7 ± 15.9	0.977
Diastolic blood pressure, mmHg	77.7 ± 11.1	77.4 ± 10.1	0.879	77.4 ± 10.8	80.4 ± 11.9	0.210
Current or former smoker	34 (58.6%)	34 (64.2%)	0.661	29 (61.7%)	31 (68.9%)	0.759
**Laboratory Results**						
Estimated GFR, mL/min/1.73 m^2	40.1 ± 7.1	40.4 ± 6.7	0.855	38.1 ± 6.3	37.2 ± 6.1	0.494
Serum creatinine, mg/dL	1.43 ± 0.17	1.42 ± 0.17	0.756	1.48 ± 0.20	1.49 ± 0.17	0.718
Serum uric acid, mg/dL	7.99 ± 0.65	7.96 ± 0.59	0.771	8.17 ± 0.64	8.00 ± 0.64	0.203
Hemoglobin A1c, %	6.0 ± 0.5	5.9 ± 0.5	0.546	6.2 ± 0.7	6.0 ± 0.6	0.119
UACR, mg/g	27.1 [8.4-102.0]	22.0 [6.8-85.7]	0.703	508.0 [181.0-1070.0]	616.0 [288.0-1100.0]	0.262
**Coexisting Conditions**						
Diabetes mellitus	15 (25.9%)	13 (24.5%)	0.872	17 (36.2%)	14 (31.1%)	0.608
Ischemic heart disease	3 (5.2%)	4 (7.5%)	0.607	2 (4.3%)	3 (6.7%)	0.610
Cerebrovascular disease	9 (15.5%)	12 (22.6%)	0.338	3 (6.4%)	5 (11.1%)	0.421
**Medications**						
ACE inhibitor and/or ARB	43 (74.1%)	39 (73.6%)	0.947	41 (87.2%)	41 (91.1%)	0.550
Statins	13 (22.4%)	21 (39.6%)	0.049	21 (44.7%)	23 (51.1%)	0.537
Antidiabetic drugs	10 (17.2%)	9 (17.0%)	0.971	16 (34.0%)	8 (17.8%)	0.076
Diuretics	6 (10.3%)	12 (22.6%)	0.079	10 (21.3%)	7 (15.6%)	0.480

Note: Values for categorical variables are given as count (percentage); values for continuous variables as mean ± standard deviation; non-normally distributed data as median [quartile 1-quartile 3]. Abbreviations: s-Cre, serum creatinine; ACE, angiotensin-converting enzyme; ARB, angiotensin receptor blocker; CKD, chronic kidney disease; GFR, glomerular filtration rate; UACR, urinary albumin-to-creatinine ratio.
